# Wound dressings – a review

**DOI:** 10.7603/s40681-015-0022-9

**Published:** 2015-11-28

**Authors:** Selvaraj Dhivya, Viswanadha Vijaya Padma, Elango Santhini

**Affiliations:** 1 Centre of Excellence for Medical Textiles, The South India Textile Research Association, 641014 Tamil Nadu Coimbatore, India; 2 Department of Biotechnology, Bharathiar University, 641044 Tamil Nadu Coimbatore, India

**Keywords:** Wound healing, Traditional dressings, Modern dressings

## Abstract

Wound healing is a dynamic and complex process which requires suitable environment to promote healing process. With the advancement in technology, more than 3000 products have been developed to treat different types of wounds by targeting various aspects of healing process. The present review traces the history of dressings from its earliest inception to the current status and also discusses the advantage and limitations of the dressing materials.

## 1. Introduction

A wound is defined as a disruption in the continuity of the epithelial lining of the skin or mucosa resulting from physical or thermal damage. According to the duration and nature of healing process, the wound is categorized as acute and chronic [1, 2]. An acute wound is an injury to the skin that occurs suddenly due to accident or surgical injury. It heals at a predictable and expected time frame usually within 8-12 weeks depending on the size, depth and the extent of damage in the epidermis and dermis layer of the skin [3, 4]. Chronic wounds on the other hand fail to progress through the normal stages of healing and cannot be repaired in an orderly and timely manner [5, 6]. Chronic wounds generally results from decubitis ulcer, leg ulcer and burns. Wound healing is a dynamic and complex process of tissue regeneration and growth progress through four different phases (i) the coagulation and haemostasis phase (immediately after injury); (ii) the inflammatory phase, (shortly after injury to tissue) during which swelling takes place; (iii) the proliferation period, where new tissues and blood vessels are formed and (iv) the maturation phase, in which remodeling of new tissues takes place [7-12]. These phases occur in an ordered manner overlapping with each other in a well-connected cascade [13, 14]. Promotion of these phases are largely depends on the wound type [15], and its associated pathological conditions and the type of dressing material. With the advancement in technology, currently, different types of wound dressing materials are available for all types of wounds. But the selection of a material for a particular wound is important to achieve faster healing. In this review, an attempt has been made to consolidate the different types of wound dressing materials and their function on healing process.

## 2. Factors affecting wound healing process

Wound healing is the result of interactions among cytokines, growth factors, blood and the extracellular matrix. The cytokines promote healing by various pathways such as stimulating the production of components of the basement membrane, preventing dehydration, increasing inflammation and the formation of granulation tissue. These pathways are affected by various local and systemic factors [16]. Local factors which includes hypothermia, pain, infection, radiation and tissue oxygen tension directly influence the characteristics of the wound where as systemic factors are the overall health or disease state of the individual that affect individual’s ability to heal [17]. In addition to these factors, poor nutrition, age and protein, vitamins and mineral deficiency can also prolongs healing times.

### 2.1 Syndromes associated with abnormal healing

Ehlers-Danlos syndrome (EDS) is a genetic connective tissue disorder characterized by defects of the major structural protein Collagen. Autosomal dominant and autosomal recessive forms of EDS equally affect males and females. Since the collagen is a major structural protein and provide elasticity to body cells and tissues, its damage results in articular hyper mobility leading to partial or complete dislocation of joints and elastic skin. Based on the defects and inheritance mode, EDS is categorized into six major subtypes and they are distinct in affecting individuals [18].

Cutis Laxa is characterized by (Lysyl oxidase) enzyme deficiency resulting in abnormality of copper metabolism leads to abnormal loose skin, muscular organ and skeletal abnormality. Wrinkled skin, particularly on the neck and mild mental retardation also characterized by this disorder. X-linked cutis laxa also called as (OHS) occipital horn syndrome, a rare disorder that was formely classified as a subtype of EDS. Cutis laxa is further classified into four genetic forms based on their pattern of inheritance. These includes sex-linked defective on X chromosome, autosomal dominant defective on autosomal chromosome and two types of autosomal recessive inheritance defective on chromosome 5 Among these types, autosomal recessive forms are more severe than other forms [19].

## 3. Characteristics of an ideal wound dressing

Based on the wound type, suitable dressing material must be used. Dressing selection should be based on its ability to a) provide or maintain moist environment b) enhance epidermal migration c) promote angiogenesis and connective tissue synthesis d) allow gas exchange between wounded tissue and environment e) maintain appropriate tissue temperature to improve the blood flow to the wound bed and enhances epidermal migration f) provide protection against bacterial infection and g) should be non-adherent to the wound and easy to remove after healing h) must provide debridement action to enhance leucocytes migration and support the accumulation of enzyme and i) must be sterile, non-toxic and non-allergic.

## 4. Wound Dressings

Wound, whether it is a minor cut or a major incision, it is important to care for it properly, part of this process includes wound dressing. Dressing is designed to be in contact with the wound, which is different from a bandage that holds the dressing in place. Historically, wet-to-dry dressings have been used extensively for wounds requiring debridement. In 1600 BC, Linen strips soaked in oil or grease covered with plasters was used to occlude wounds. Clay tablets were used for the treatment of wounds by Mesopotamian origin from about 2500 BCE. They cleaned wounds with water or milk prior to dressing with honey or resin. Wine or vinegar usage for cleaning the wounds with honey, oil and wine as further treatment was followed by Hippocrates of ancient Greece in 460- 370 BCE. They used wool boiled in water or wine as a bandage [20]. There was a major breakthrough in the antiseptic technique during the 19^th^ century, antibiotics were introduced to control infections and decrease mortality. Modern wound dressing arrival was in 20^th^ century [21].

When the wound is closed with dressing they are continuously exposed to proteinases, chemotactic, complement & growth factors, which is lost in the wound exposed. So during late 20^th^ century, production of occlusive dressing began to protect and provide moist environment to wound. These dressings helps in faster re-epithelialization, collagen synthesis, promotes angiogenesis by creating hypoxia to the wound bed and decreases wound bed pH which leads to decrease in the wound infection [22]. Woven absorbent cotton gauze was used in 1891. Until the mid 1900’s, it was firmly believed that wounds healed more quickly if kept dry and uncovered whereas ‘closed wounds heal more quickly than open wound’ written in an Egyptian medical text -Edwin smith surgical papyrus in 1615 BC. Oscar Gilje in 1948 describes moist chamber effect for healing ulcers. In the mid 1980’s, the first modern wound dressing were introduced which delivered important characteristics providing moisture and absorbing fluids (e.g. polyurethane foams, hydrocolloids, iodine-containing gels). During the mid 1990’s, synthetic wound dressings expanded into various group of products which includes hydrogels, hydrocolloids, alginates, synthetic foam dressing, silicone meshes, tissue adhesives, vapor-permeable adhesive films and silver/collagen containing dressing.

### 4.1. Traditional wound dressing

Traditional wound dressing products including gauze, lint, plasters, bandages (natural or synthetic) and cotton wool are dry and used as primary or secondary dressings for protecting the wound from contaminations [30]. Gauze dressings made out of woven and non woven fibres of cotton, rayon, polyesters afford some sort of protection against bacterial infection. Some sterile gauze pads are used for absorbing exudates and fluid in an open wound with the help of fibres in these dressings. These dressings require frequent changing to protect from maceration of healthy tissues. Gauze dressings are less cost effective. Due to excessive wound drainage, dressings become moistened and tend to become adherent to the wound making it painful when removing. Bandages made out of natural cotton wool and cellulose or synthetic bandages made out of polyamide materials perform different functions. For instance, cotton bandages are used for retention of light dressings, high compression bandages and short stretch compression bandages provide sustained compression in case of venous ulcers. Xeroform™ (non-occlusive dressing) is petrolatum gauze with 3% of Bismuth tribromophenate used for non-exudating to slight exudating wounds. Tulle dressings such as Bactigras, Jelonet, Paratulle are some examples of tulle dressings commercially available as impregnated dressings with paraffin and suitable for superficial clean wound. Generally traditional dressings are indicated for the clean and dry wounds with mild exudate levels or used as secondary dressings. Since traditional dressings fail to provide moist environment to the wound they have been replaced by modern dressings with more advanced formulations [30].

### 4.2. Modern wound dressing

Modern wound dressing have been developed to facilitate the function of the wound rather than just to cover it. These dressings are focused to keep the wound from dehydration and promote healing. Based on the cause and type of wound, numerous products are available in the market, making the selection a very difficult task. Modern wound dressings are usually based on synthetic polymers and are classified as passive, interactive and bioactive products. Passive products are non-occlusive, such as gauze and tulle dressings, used to cover the wound to restore its function underneath. Interactive dressings are semi-occlusive or occlusive, available in the forms of films, foam, hydrogel and hydrocolloids. These dressings act as a barrier against penetration of bacteria to the wound environment [11-14].

#### 4.2.1. Semi-permeable film dressings

These dressings are composed of transparent and adherent polyurethane which permits transmission of water vapor, O_2_ and CO_2_ from the wound and it also provides autolytic debridement of eschar and impermeable to bacteria [23]. Initially, films were made from nylon derivatives with an adhesive polyethylene frames as the support which made them occlusive. Originally nylon derived film dressings were not used for highly exudating wounds due to their limited absorption capacity and caused maceration of the wound and the healthy tissues around the wound [24]. But, these dressings are highly elastic and flexible, and can conform to any shape and do not require additional tapping. Inspection of wound closure is also possible without removal of wound dressing because of transparent films. Hence these dressings are recommended for epithelializing wound, superficial wound and shallow wound with low exudates, e.g. Opsite™, Tegaderm™, Biooclusive ™. Commercially available film dressings differ in terms of their vapour permeability, adhesive characteristics, conformability and extensibility [25].

#### 4.2.2. Semi-permeable foam dressings

Foam dressings are made up of hydrophobic and hydrophilic foam with adhesive borders sometimes [26]. The hydrophobic properties of outer layer protect from the liquid but allow gaseous exchange and water vapor. Silicone-based rubber foam (silastic) molds and contours to wound shape. Foam has capability of absorbing varying quantities of wound drainage depending upon the wound thickness. Adhesive and non adhesive foam dressings are available. Foam dressings are suitable for lower leg ulcers and moderate to highly exudating wounds, also indicated for granulating wounds. They are generally used as primary dressings for absorption and secondary dressings are not required due to their high absorbancy and moisture vapour permeability [27, 28]. Disadvantage of foam dressing is requiring frequent dressing and is not suitable for low exudating wounds, dry wounds and dry scars as they depend on exudates for its healing [28] e.g. Lyofoam™, Allevyn™ and Tielle™.

#### 4.2.3. Hydrogels dressing

Hydrogels are insoluble hydrophilic materials made from synthetic polymers such as poly (methacrylates) and polyvinyl pyrrolidine. The high water content of hydrogels (70-90 %) helps granulation tissues and epithelium in a moist environment. Soft elastic property of hydrogels provides easy application and removal after wound is healed without any damage. Temperature of cutaneous wounds is decreased by hydrogels providing soothing and cooling effect. Hydrogels are used for dry chronic wounds, necrotic wounds, pressure ulcers and burn wounds. Morgan [27] has reported that except infected and heavy drainage wounds, hydrogel dressings are suitable for all four stages of wound healing. Hydrogel dressings are non irritant, non reactive with biological tissue and permeable to metabolites. Many researchers have reported that hydrogel dressings are used to treat chronic leg ulcers. Difficulties of hydrogel dressings are exudate accumulation leads to maceration and bacterial proliferation that produces foul smell in wounds. Besides, low mechanical strength of hydrogels making it difficult to handle [29]. Some examples of hydrogels are Intrasite™, Nu-gel™, Aquaform™ polymers, sheet dressings, impregnated gauze and water-based gels.

#### 4.2.4. Hydrocolloid dressing

Hydrocolloid dressings are among the most widely used interactive dressings and are consist of two layers, inner colloidal layer and outer water- impermeable layer. These dressings are made up of the combination of gel forming agents (carboxymethylcellulose, gelatin and pectin) with other materials such as elastomers and adhesives [30]. Hydrocolloids are permeable to water vapor but impermeable to bacteria and also have the properties of debridement and absorb wound exudates [31]. They are used on light to moderately exudating wounds such as pressure sores, minor burn wounds and traumatic wounds. These dressings are also recommended for paediatric wound care management, as they do not cause pain on removal [32]. When this hydrocolloids contact with the wound exudate they form gels and provide moist environment that helps in protection of granulation tissue by absorbing and retaining exudates. Granuflex™, Comfeel™, Tegasorb™ are available in the form of sheets or thin films. Disadvantage of hydrocolloids are they are not indicated for neuropathic ulcers or highly exudating wounds, also they are mostly used as a secondary dressings [30].

#### 4.2.5. Alginate dressing

Alginate dressings are made from the sodium and calcium salts comprising mannuronic and guluronic acid units. Absorbent and biodegradable alginates are derived from seaweed. Absorption capability is achieved by strong hydrophilic gel formation, which limits wound exudates and minimizes bacterial contamination. Even though some studies have reported that alginate inhibits keratinocytes migration, Thomas *et al*., [33] have reported that alginates accelerate healing process by activating macrophages to produce TNF-α which initiates inflammatory signals. Once alginate dressings are applied to the wound, ions present in the alginate are exchanged with blood to form a protective film. Alginate dressings are suitable for moderate to heavy drainage wounds and not suggested for dry wound, third degree burn wound and severe wounds with exposed bone. Also these dressings require secondary dressings because it could dehydrate the wound which delay healing. Sorbsan™, Kaltostat™, Algisite™ are some alginate dressings commercially available [30].

### 4.3. Bioactive wound dressings

The last type of modern wound dressing is bioactive dressings and is produced from biomaterials which play an important role in healing process. These dressings are known for their biocompatibility, biodegradability and non-toxic nature and are derived generally from natural tissues or artificial sources [34] such as collagen [35], hyaluronic acid [36], chitosan [37], alginate and elastin. Polymers of these materials are used alone or in combination depending on the nature and type of wound. Biological dressings are sometimes incorporated with growth factors and antimicrobials to enhance wound healing process.

Collagen, a major structural protein has been discussed by many researchers for their active role in natural healing process [35, 38, 39]. Collagen initiates fibroblast formation and accelerates endothelial migration upon contact with wound tissue [40]. Hyaluronic acid (HA) is a glycoaminoglycan component of extra cellular matrix (ECM) with unique biological and physicochemical features. Similar to collagen, HA also biocompatible, biodegradable and lack immunogenicity naturally [41]. Chitosan promotes the formation of granulation tissue during the proliferative stage of wound healing [42]. When compared to other dressings, biological dressings are reported to be more superior to other types of dressings.

### 4.4. Tissue engineered skin substitutes

Human skin or dermal equivalent (HSE) has two types of tissueengineered substitutes available, one mimics the layer of skin composed of Keratinocytes and fibroblast on collagen matrix (Cell containing matrix). Second contains only the dermal elements with fibroblast on collagen matrix (Acellular matrix). Major mechanism of HSE is to secrete and stimulate wound growth factor by which epithelialization is achieved. Bioengineered are capable of adapting to their environment so that they are able to release growth factors and cytokines incorporated in dressings. Bioengineered dressings are suitable for Diabetic foot ulcer and venous leg ulcer. Apligraf is a FDA approved skin equivalent substitute consists of keratinocytes and fibroblast-seeded collagen for venous ulcers. Some skin substitutes commercially available include, Alloderm™ composed of normal human fibroblasts with all cellular materials removed and Integra™ artificial skin consists of collagen/ chondroitin 6 sulphate matrix overlaid with a thin silicone sheet. Other few substitutes are Laserskin™, Biobrane ™, Bioseed™, and Hyalograft3-DTM.

### 4.5. Medicated dressings

Medicated dressings incorporated drugs plays an important role in the healing process directly or indirectly by removal of necrotic tissues. This has been achieved by cleaning or debriding agents for necrotic tissue, antimicrobials which prevents infection and promotes tissue regeneration. Some commonly incorporated compounds include antimicrobial agents, growth factors and enzymes. Commercially available antimicrobial dressings include Cutisorb™. Silver impregnated dressings available are Fibrous hydrocolloid, Polyurethane foam film and silicone gels. Antiseptic Iodine dressing acts on bacterial cells via oxidative degradation of cell components by interrupting the function of protein, which is widely effective against pathogen. Prolong usage of iodine leads to skin irritation and staining [43]. The purpose of antimicrobials is mainly to prevent or combat infections especially for diabetic foot ulcers.

Normal tissue repair process in the body is controlled by cellular activities caused by growth factors that are naturally present in our body. In case of chronic wounds, growth factors and cells are arrested in the wound bed within the clots that affects the healing process. So exogenous application of growth factors benefits the wound healing process and this was proved by numerous studies. Among the different growth factors, platelet derived growth factor (PDGF) is the most commonly used growth factor which promotes chemotactic recruitment and proliferation of cells and increasing angiogenesis. Besides, PDGF, fibroblast growth factor (FGF), epidermal growth factor (EGF), and autologous platelet thrombin are also studied extensively for their application in healing process. Among which, PDGF and EGF are approved by FDA for human application.

Enzymatic debridement of necrotic tissues without harming healthy tissue is also a crucial part to promote normal healing process. Papain and collagenase based ointments are currently used to digest necrotic tissue. Collagenase acts on the collagen by attacking native collagen and gentle on viable collagen by gradual breakdown of tissue whereas papain attacks cystein residue and associated with inflammatory response. DebridaceTM is a commercially available dressing which increases proteolytic action.

### 4.6. Composite dressing

Composite dressings are versatile and convenient for both partial and full thickness wounds. A composite or combination dressings dressings has multiple layers and each layer is physiologically distinct. Most of the composite dressings possess three layers. Composite dressings may also include an adhesive border of non-woven fabric tape or transparent film. They can function as either a primary or a secondary dressing on a wide variety of wounds and may be used with topical medications. Outer most layer protect the wound from infection, middle layer usually composed of absorptive material which maintains moisture environment and assist autolytic debridement, bottom layer composed of non adherent material which prevents from sticking to young granulating tissues. Composite dressings have less flexibility and they are more expensive [44].

## 5. Conclusion

Currently more than 3000 types of dressings are available in the market making the physician to address all aspects of wound care. But still there is no superior product that heals chronic wounds like venous leg ulcers, diabetic wound and pressure ulcers which often fail to achieve complete healing. Hence developing a dressing material that addresses the major interfering factors of normal healing process will help patients and wound care practitioners largely.
